# (*E*)-2-[(2,4-Dichloro­phen­yl)imino­methyl]-6-methyl­phenol

**DOI:** 10.1107/S1600536809009684

**Published:** 2009-03-19

**Authors:** Zarife Sibel Şahin, Şamil Işık, Ferda Erşahin, Erbil Ağar

**Affiliations:** a Department of Physics, Faculty of Arts and Sciences, Ondokuz Mayıs University, Kurupelit, TR-55139 Samsun, Turkey; b Department of Chemistry, Faculty of Arts and Sciences, Ondokuz Mayıs University, TR-55139 Samsun, Turkey

## Abstract

The title compound, C_14_H_11_Cl_2_NO, is a Schiff base which adopts the phenol–imine tautomeric form in the solid state. There are two mol­ecules in the asymmetric unit. Head-to-tail π–π inter­actions [centroid-to-centroid distances of 3.682 (2), 3.708 (2), 3.904 (2) and 3.910 (2) Å] between adjacent mol­ecules produce two symmetry-independent infinite chains running along the *b* axis.

## Related literature

For the biological properties of Schiff bases, see: Lozier *et al.* (1975 ▶). For Schiff base tautomerism, see: Şahin *et al.* (2005 ▶); Hadjoudis *et al.* (1987 ▶). For the structure of a similar compound, see: Karataş *et al.* (2005 ▶). For the classification of hydrogen-bonding patterns, see: Bernstein *et al.* (1995 ▶).
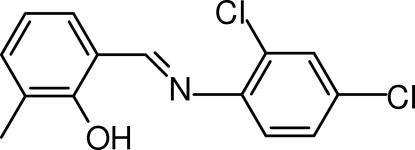


## Experimental

### 

#### Crystal data

C_14_H_11_Cl_2_NO
*M* *_r_* = 280.14
Monoclinic, 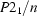

*a* = 19.981 (2) Å
*b* = 7.1473 (6) Å
*c* = 20.057 (4) Å
β = 114.913 (11)°
*V* = 2597.8 (7) Å^3^
*Z* = 8
Mo *K*α radiation
μ = 0.48 mm^−1^
*T* = 296 K
0.45 × 0.21 × 0.11 mm

#### Data collection

Stoe IPDSII diffractometer
Absorption correction: integration (*X-RED32*; Stoe & Cie, 2002 ▶) *T*_min_ = 0.903, *T*_max_ = 0.953
30840 measured reflections
5370 independent reflections
3188 reflections with *I* > 2σ(*I*)
*R*_int_ = 0.056

#### Refinement

*R*[*F* ^2^ > 2σ(*F* ^2^)] = 0.040
*wR*(*F* ^2^) = 0.106
*S* = 0.88
5370 reflections
334 parameters
2 restraints
H atoms treated by a mixture of independent and constrained refinement
Δρ_max_ = 0.40 e Å^−3^
Δρ_min_ = −0.40 e Å^−3^

### 

Data collection: *X-AREA* (Stoe & Cie, 2002 ▶); cell refinement: *X-AREA*; data reduction: *X-RED32* (Stoe & Cie, 2002 ▶); program(s) used to solve structure: *SHELXS97* (Sheldrick, 2008 ▶); program(s) used to refine structure: *SHELXL97* (Sheldrick, 2008 ▶); molecular graphics: *ORTEP-3 for Windows* (Farrugia, 1997 ▶); software used to prepare material for publication: *WinGX* (Farrugia, 1999 ▶) and *PLATON* (Spek, 2009 ▶).

## Supplementary Material

Crystal structure: contains datablocks I, global. DOI: 10.1107/S1600536809009684/ya2087sup1.cif

Structure factors: contains datablocks I. DOI: 10.1107/S1600536809009684/ya2087Isup2.hkl

Additional supplementary materials:  crystallographic information; 3D view; checkCIF report

## Figures and Tables

**Table 1:** Hydrogen-bond geometry (Å, °)

*D*—H⋯*A*	*D*—H	H⋯*A*	*D*⋯*A*	*D*—H⋯*A*
O1*A*—H1*A*⋯N1*A*	0.834 (17)	1.86 (2)	2.603 (2)	149 (3)
O1*B*—H1*B*⋯N1*B*	0.798 (17)	1.86 (2)	2.599 (2)	155 (3)

**Fig. 1.**
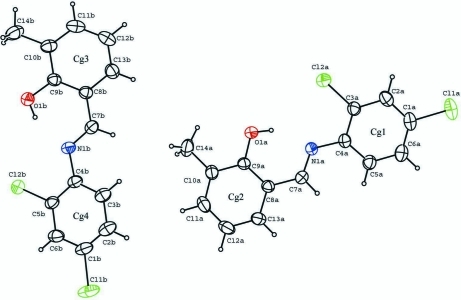
Molecular structure of the title compound, showing the atom-numbering scheme. Displacement ellipsoids are drawn at the 30% probability.

**Fig. 2.**
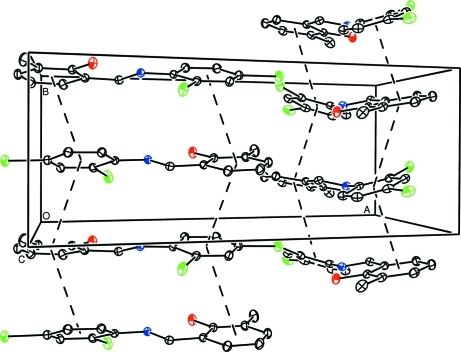
Infinite chains formed due to the π-π interactions in the crystal packing of the title compound; the packing is viewed down the c axis.

## supplementary crystallographic information

### Comment

Schiff bases often exhibit various biological activities and in many cases were shown to have antibacterial, anticancer, anti-inflammatory and antitoxic properties.(Lozier *et al.*, 1975). There are two types of intramolecular hydrogen bonds in Schiff bases, which may be stabilized either in keto-amine (N–H···O hydrogen bond)(Şahin *et al.*, 2005) or phenol-imine (N···H–O hydrogen bond) tautomeric forms (Hadjoudis *et al.*, 1987). The present X-ray investigation shows that the title compound exists in the phenol-imine form.

There are two symmetry independent molecules in the crystal of the the title cmpound (Fig. 1). The N1A—C7A and N1B—C7B bond lengths are consistent with the double-bond character of these bonds (Table 1). The title compound is similar to that reported by Karataş *et al.*, 2005. However, while the molecule of the latter compound is almost planar, the title compound, which has a Me substituent, significantly deviates from planarity with the dihedral angles of 30.40 (5)° and 27.84 (5)° for the *Cg*1 / *Cg*2, and *Cg*3 / *Cg*4 plane pairs respectively. The C4A—N1A—C7A—C8A and C4B—N1B—C7B—C8B torsion angles are 178.4 (2)° and 177.5 (2)°; the intramolecular O–H···N hydrogen bonds produce S(6) rings within each of the two molecules (Bernstein *et al.*,1995).

The crystal packing of the title compound features four symmetry independent π-π interactions (Fig. 2). The perpendicular distances from *Cg*1 to *Cg*2^i^ [symmetry code: (i) = 3/2 - *x*, -1/2 + *y*, 1/2 - *z*] and from *Cg*1 to *Cg*2^ii^[symmetry code: (ii) = 3/2 - *x*, 1/2 + *y*, 1/2 - *z*] are 3.449 (2) Å and 3.537 (2) Å respectively (see *PLATON* description for exact definition of the parameters; Spek, 2009). The centroid-to-centroid distances are 3.682 (2) Å (*Cg*1 to *Cg*2^i^) and 3.910 (2) Å (*Cg*1 to *Cg*2^ii^). Similarly, ring *Cg*4 is oriented in such a way that the perpendicular distances for *Cg*4—*Cg*3^iii^ and *Cg*4—*Cg*3^iv^ are 3.500 (2) Å and 3.492 (2) Å [symmetry codes: (iii) = 1/2 - *x*, -1/2 + *y*, 1/2 - *z*; (iv) = 1/2 - *x*, 1/2 + *y*, 1/2 - *z*], and the distances between the ring centroids are 3.904 (2) Å (*Cg*4 to *Cg*3^iii^) and 3.708 (2) Å (*Cg*4 to *Cg*3^iv^).

### Experimental

The solution of 3-methylsalicyladehyde (0.1 g, 0.82 mmol) in 20 ml e thanol was mixed with the 20 ml of the ethanol solution of 2,4-dichloroaniline (0.13 g 0.82 mmol). The reaction mixture was then refluxed for 2 hrs under stirring. The single crystals of the title compound suitable for X-ray analysis were obtained by slow evaporation from ethanol (yield, %: 62; m.p. 370–372 K).

### Refinement

The H1A and H1B atoms were located in a difference map and subsequently refined subject to a *DFIX* (*SHELXL97*; Sheldrick, 2008) restraint of O—H=0.82 (2) Å. All other H atoms were placed in calculated positions and constrained to ride on their parents atoms, with C—H=0.93–0.96 Å and *U*_iso_(H)=1.2*U*_eq_(C) [1.5*U*_eq_(C) for the methyl H atoms].

### Crystal data

**Table:**

C_14_H_11_Cl_2_NO	*F*(000) = 1152
*M**_r_* = 280.14	*D*_x_ = 1.433 Mg m^−^^3^
Monoclinic, *P*2_1_/*n*	Mo *K*α radiation, λ = 0.71073 Å
Hall symbol: -P 2yn	Cell parameters from 24378 reflections
*a* = 19.981 (2) Å	θ = 1.9–27.8°
*b* = 7.1473 (6) Å	µ = 0.48 mm^−^^1^
*c* = 20.057 (4) Å	*T* = 296 K
β = 114.913 (11)°	Prism, yellow
*V* = 2597.8 (7) Å^3^	0.45 × 0.21 × 0.11 mm
*Z* = 8	

### Data collection

**Table:**

Stoe IPDSII diffractometer	5370 independent reflections
Radiation source: fine-focus sealed tube	3188 reflections with *I* > 2σ(*I*)
graphite	*R*_int_ = 0.056
Detector resolution: 6.67 pixels mm^-1^	θ_max_ = 26.5°, θ_min_ = 1.9°
ω scans	*h* = −25→25
Absorption correction: integration (*X-RED32*; Stoe & Cie, 2002)	*k* = −8→8
*T*_min_ = 0.903, *T*_max_ = 0.953	*l* = −25→25
30840 measured reflections	

### Refinement

**Table:**

Refinement on *F*^2^	Secondary atom site location: difference Fourier map
Least-squares matrix: full	Hydrogen site location: inferred from neighbouring sites
*R*[*F*^2^ > 2σ(*F*^2^)] = 0.040	H atoms treated by a mixture of independent and constrained refinement
*wR*(*F*^2^) = 0.106	*w* = 1/[σ^2^(*F*_o_^2^) + (0.0615*P*)^2^] where *P* = (*F*_o_^2^ + 2*F*_c_^2^)/3
*S* = 0.88	(Δ/σ)_max_ = 0.002
5370 reflections	Δρ_max_ = 0.40 e Å^−^^3^
334 parameters	Δρ_min_ = −0.40 e Å^−^^3^
2 restraints	Extinction correction: *SHELXL97* (Sheldrick, 2008), Fc^*^=kFc[1+0.001xFc^2^λ^3^/sin(2θ)]^-1/4^
Primary atom site location: structure-invariant direct methods	Extinction coefficient: 0.0055 (5)

### Special details

**Table:**

Geometry. All e.s.d.'s (except the e.s.d. in the dihedral angle between two l.s. planes) are estimated using the full covariance matrix. The cell e.s.d.'s are taken into account individually in the estimation of e.s.d.'s in distances, angles and torsion angles; correlations between e.s.d.'s in cell parameters are only used when they are defined by crystal symmetry. An approximate (isotropic) treatment of cell e.s.d.'s is used for estimating e.s.d.'s involving l.s. planes.
Refinement. Refinement of *F*^2^ against ALL reflections. The weighted *R*-factor *wR* and goodness of fit *S* are based on *F*^2^, conventional *R*-factors *R* are based on *F*, with *F* set to zero for negative *F*^2^. The threshold expression of *F*^2^ > σ(*F*^2^) is used only for calculating *R*-factors(gt) *etc*. and is not relevant to the choice of reflections for refinement. *R*-factors based on *F*^2^ are statistically about twice as large as those based on *F*, and *R*- factors based on ALL data will be even larger.

### Fractional atomic coordinates and isotropic or equivalent isotropic displacement parameters (Å^2^)

**Table:**

	*x*	*y*	*z*	*U*_iso_*/*U*_eq_	
C1A	0.03154 (13)	0.4396 (4)	0.67838 (15)	0.0634 (6)	
C1B	0.81914 (17)	0.2780 (3)	0.96769 (12)	0.0640 (7)	
C2A	0.05942 (13)	0.3951 (4)	0.62832 (13)	0.0621 (6)	
H2A	0.0279	0.3652	0.5801	0.075*	
C2B	0.74914 (17)	0.2265 (4)	0.92203 (13)	0.0683 (7)	
H2B	0.7163	0.1896	0.9413	0.082*	
C3A	0.13455 (12)	0.3954 (3)	0.65054 (11)	0.0527 (5)	
C3B	0.72734 (15)	0.2291 (3)	0.84732 (12)	0.0620 (6)	
H3B	0.6795	0.1937	0.8163	0.074*	
C4A	0.18332 (11)	0.4388 (3)	0.72239 (11)	0.0475 (5)	
C4B	0.77533 (13)	0.2836 (3)	0.81725 (11)	0.0508 (5)	
C5A	0.15257 (13)	0.4858 (3)	0.77090 (12)	0.0573 (6)	
H5A	0.1836	0.5182	0.8190	0.069*	
C5B	0.84670 (13)	0.3341 (3)	0.86513 (11)	0.0537 (6)	
C6A	0.07801 (14)	0.4855 (4)	0.74967 (14)	0.0651 (7)	
H6A	0.0588	0.5162	0.7832	0.078*	
C6B	0.86936 (15)	0.3331 (4)	0.94063 (11)	0.0620 (6)	
H6B	0.9170	0.3684	0.9723	0.074*	
C7A	0.30675 (12)	0.4072 (3)	0.80567 (11)	0.0482 (5)	
H7A	0.2903	0.3737	0.8411	0.058*	
C7B	0.69131 (13)	0.3133 (3)	0.69404 (11)	0.0518 (5)	
H7B	0.6559	0.3458	0.7107	0.062*	
C8A	0.38465 (11)	0.4160 (3)	0.82605 (10)	0.0463 (5)	
C8B	0.67009 (12)	0.3030 (3)	0.61578 (11)	0.0487 (5)	
C9A	0.41332 (12)	0.4551 (3)	0.77458 (10)	0.0471 (5)	
C9B	0.72176 (12)	0.2636 (3)	0.58774 (10)	0.0482 (5)	
C10A	0.48888 (12)	0.4609 (3)	0.79476 (12)	0.0562 (6)	
C10B	0.70102 (14)	0.2580 (3)	0.51193 (12)	0.0579 (6)	
C11A	0.53546 (13)	0.4309 (4)	0.86787 (14)	0.0659 (7)	
H11A	0.5862	0.4369	0.8826	0.079*	
C11B	0.62790 (16)	0.2882 (4)	0.46609 (12)	0.0696 (7)	
H11B	0.6131	0.2834	0.4155	0.084*	
C12A	0.50878 (14)	0.3922 (4)	0.91977 (13)	0.0710 (7)	
H12A	0.5414	0.3723	0.9686	0.085*	
C12B	0.57587 (15)	0.3253 (4)	0.49248 (13)	0.0733 (7)	
H12B	0.5268	0.3445	0.4600	0.088*	
C13A	0.43479 (13)	0.3833 (4)	0.89929 (11)	0.0607 (6)	
H13A	0.4171	0.3552	0.9342	0.073*	
C13B	0.59656 (13)	0.3339 (4)	0.56658 (13)	0.0629 (6)	
H13B	0.5616	0.3605	0.5845	0.076*	
C14A	0.51781 (15)	0.4976 (5)	0.73832 (16)	0.0848 (9)	
H10A	0.5484	0.6073	0.7518	0.127*	
H10B	0.4772	0.5167	0.6913	0.127*	
H10C	0.5464	0.3923	0.7357	0.127*	
C14B	0.75762 (17)	0.2210 (4)	0.48301 (14)	0.0827 (9)	
H10D	0.7347	0.2258	0.4303	0.124*	
H10E	0.7788	0.0994	0.4987	0.124*	
H10F	0.7957	0.3141	0.5015	0.124*	
N1A	0.25916 (9)	0.4438 (2)	0.74044 (9)	0.0467 (4)	
N1B	0.75702 (11)	0.2794 (3)	0.74117 (9)	0.0504 (4)	
O1A	0.36837 (9)	0.4854 (3)	0.70326 (8)	0.0620 (5)	
H1A	0.3248 (10)	0.476 (4)	0.6980 (15)	0.083 (9)*	
O1B	0.79332 (9)	0.2323 (3)	0.63215 (9)	0.0611 (4)	
H1B	0.7955 (17)	0.240 (4)	0.6727 (11)	0.093 (10)*	
Cl1A	−0.06295 (4)	0.43590 (12)	0.65100 (5)	0.0885 (3)	
Cl1B	0.84855 (5)	0.26781 (11)	1.06241 (3)	0.0916 (3)	
Cl2A	0.17044 (4)	0.33856 (12)	0.58800 (3)	0.0787 (2)	
Cl2B	0.90870 (3)	0.40133 (11)	0.82999 (3)	0.0724 (2)	

### Atomic displacement parameters (Å^2^)

**Table:**

	*U*^11^	*U*^22^	*U*^33^	*U*^12^	*U*^13^	*U*^23^
C1A	0.0444 (13)	0.0550 (16)	0.0929 (18)	0.0012 (12)	0.0309 (13)	0.0058 (13)
C1B	0.102 (2)	0.0526 (15)	0.0446 (11)	0.0191 (15)	0.0379 (14)	0.0065 (11)
C2A	0.0472 (13)	0.0630 (17)	0.0672 (14)	−0.0081 (12)	0.0154 (11)	0.0032 (12)
C2B	0.098 (2)	0.0608 (17)	0.0586 (13)	0.0009 (15)	0.0457 (15)	0.0054 (12)
C3A	0.0484 (13)	0.0558 (14)	0.0540 (12)	−0.0056 (11)	0.0217 (10)	0.0024 (10)
C3B	0.0789 (17)	0.0586 (15)	0.0553 (12)	0.0000 (13)	0.0350 (13)	0.0058 (11)
C4A	0.0431 (11)	0.0440 (13)	0.0557 (12)	0.0006 (10)	0.0210 (10)	0.0038 (10)
C4B	0.0681 (15)	0.0443 (13)	0.0428 (10)	0.0102 (11)	0.0259 (11)	0.0030 (9)
C5A	0.0538 (14)	0.0601 (15)	0.0620 (13)	0.0029 (12)	0.0284 (11)	−0.0021 (11)
C5B	0.0669 (15)	0.0527 (14)	0.0456 (11)	0.0156 (12)	0.0279 (11)	0.0027 (10)
C6A	0.0586 (15)	0.0636 (17)	0.0840 (17)	0.0035 (13)	0.0407 (14)	−0.0023 (13)
C6B	0.0773 (17)	0.0599 (15)	0.0432 (11)	0.0171 (14)	0.0199 (11)	−0.0011 (10)
C7A	0.0519 (13)	0.0489 (14)	0.0484 (11)	−0.0002 (11)	0.0258 (10)	−0.0014 (9)
C7B	0.0592 (14)	0.0500 (14)	0.0518 (12)	0.0047 (11)	0.0289 (11)	0.0034 (10)
C8A	0.0452 (12)	0.0481 (13)	0.0428 (10)	0.0003 (10)	0.0158 (9)	−0.0047 (9)
C8B	0.0510 (13)	0.0481 (13)	0.0454 (10)	−0.0025 (11)	0.0188 (10)	0.0024 (9)
C9A	0.0450 (12)	0.0519 (14)	0.0423 (10)	−0.0005 (10)	0.0165 (9)	−0.0078 (9)
C9B	0.0488 (12)	0.0496 (13)	0.0435 (10)	−0.0090 (11)	0.0166 (10)	−0.0012 (9)
C10A	0.0468 (13)	0.0602 (16)	0.0618 (13)	−0.0023 (12)	0.0230 (11)	−0.0112 (11)
C10B	0.0729 (16)	0.0553 (15)	0.0465 (11)	−0.0142 (13)	0.0262 (11)	−0.0025 (10)
C11A	0.0419 (13)	0.0701 (18)	0.0743 (16)	0.0005 (12)	0.0134 (12)	−0.0159 (13)
C11B	0.0843 (19)	0.0688 (18)	0.0425 (11)	−0.0127 (15)	0.0139 (13)	0.0004 (11)
C12A	0.0587 (16)	0.083 (2)	0.0504 (13)	0.0082 (15)	0.0027 (12)	−0.0024 (12)
C12B	0.0631 (16)	0.0766 (19)	0.0566 (14)	0.0003 (15)	0.0020 (13)	0.0070 (13)
C13A	0.0575 (15)	0.0734 (17)	0.0441 (11)	0.0071 (13)	0.0145 (11)	0.0024 (11)
C13B	0.0550 (15)	0.0663 (17)	0.0640 (14)	0.0043 (13)	0.0217 (12)	0.0043 (12)
C14A	0.0615 (17)	0.115 (3)	0.0924 (19)	−0.0119 (17)	0.0470 (15)	−0.0137 (18)
C14B	0.098 (2)	0.103 (2)	0.0605 (14)	−0.0235 (19)	0.0466 (15)	−0.0126 (15)
N1A	0.0412 (9)	0.0507 (11)	0.0474 (9)	−0.0005 (9)	0.0179 (8)	−0.0015 (8)
N1B	0.0604 (12)	0.0514 (11)	0.0403 (8)	0.0030 (10)	0.0220 (9)	0.0018 (8)
O1A	0.0509 (10)	0.0930 (13)	0.0418 (8)	−0.0039 (9)	0.0190 (7)	0.0000 (8)
O1B	0.0493 (9)	0.0849 (13)	0.0507 (9)	−0.0049 (9)	0.0226 (8)	−0.0047 (8)
Cl1A	0.0454 (3)	0.0874 (6)	0.1318 (6)	−0.0008 (4)	0.0364 (4)	0.0067 (5)
Cl1B	0.1469 (7)	0.0863 (5)	0.0449 (3)	0.0213 (5)	0.0437 (4)	0.0063 (3)
Cl2A	0.0639 (4)	0.1209 (6)	0.0530 (3)	−0.0176 (4)	0.0263 (3)	−0.0127 (3)
Cl2B	0.0588 (4)	0.1028 (6)	0.0590 (3)	0.0109 (4)	0.0281 (3)	0.0000 (3)

### Geometric parameters (Å, °)

**Table:**

C1A—C2A	1.375 (3)	C8A—C9A	1.404 (3)
C1A—C6A	1.377 (4)	C8A—C13A	1.406 (3)
C1A—Cl1A	1.730 (2)	C8B—C9B	1.397 (3)
C1B—C2B	1.361 (4)	C8B—C13B	1.400 (3)
C1B—C6B	1.384 (4)	C9A—O1A	1.348 (2)
C1B—Cl1B	1.738 (2)	C9A—C10A	1.389 (3)
C2A—C3A	1.374 (3)	C9B—O1B	1.347 (3)
C2A—H2A	0.9300	C9B—C10B	1.398 (3)
C2B—C3B	1.373 (3)	C10A—C11A	1.383 (3)
C2B—H2B	0.9300	C10A—C14A	1.495 (3)
C3A—C4A	1.393 (3)	C10B—C11B	1.377 (4)
C3A—Cl2A	1.735 (2)	C10B—C14B	1.496 (4)
C3B—C4B	1.387 (3)	C11A—C12A	1.383 (4)
C3B—H3B	0.9300	C11A—H11A	0.9300
C4A—C5A	1.393 (3)	C11B—C12B	1.377 (4)
C4A—N1A	1.402 (3)	C11B—H11B	0.9300
C4B—C5B	1.390 (3)	C12A—C13A	1.359 (3)
C4B—N1B	1.412 (2)	C12A—H12A	0.9300
C5A—C6A	1.366 (3)	C12B—C13B	1.366 (3)
C5A—H5A	0.9300	C12B—H12B	0.9300
C5B—C6B	1.386 (3)	C13A—H13A	0.9300
C5B—Cl2B	1.731 (2)	C13B—H13B	0.9300
C6A—H6A	0.9300	C14A—H10A	0.9600
C6B—H6B	0.9300	C14A—H10B	0.9600
C7A—N1A	1.279 (3)	C14A—H10C	0.9600
C7A—C8A	1.435 (3)	C14B—H10D	0.9600
C7A—H7A	0.9300	C14B—H10E	0.9600
C7B—N1B	1.277 (3)	C14B—H10F	0.9600
C7B—C8B	1.445 (3)	O1A—H1A	0.834 (17)
C7B—H7B	0.9300	O1B—H1B	0.798 (17)
			
C2A—C1A—C6A	120.7 (2)	C13B—C8B—C7B	119.9 (2)
C2A—C1A—Cl1A	119.2 (2)	O1A—C9A—C10A	117.44 (18)
C6A—C1A—Cl1A	120.1 (2)	O1A—C9A—C8A	121.10 (19)
C2B—C1B—C6B	121.5 (2)	C10A—C9A—C8A	121.45 (19)
C2B—C1B—Cl1B	120.2 (2)	O1B—C9B—C8B	121.73 (18)
C6B—C1B—Cl1B	118.3 (2)	O1B—C9B—C10B	117.4 (2)
C3A—C2A—C1A	119.1 (2)	C8B—C9B—C10B	120.9 (2)
C3A—C2A—H2A	120.5	C11A—C10A—C9A	117.9 (2)
C1A—C2A—H2A	120.5	C11A—C10A—C14A	121.9 (2)
C1B—C2B—C3B	119.6 (2)	C9A—C10A—C14A	120.3 (2)
C1B—C2B—H2B	120.2	C11B—C10B—C9B	117.8 (2)
C3B—C2B—H2B	120.2	C11B—C10B—C14B	122.1 (2)
C2A—C3A—C4A	121.9 (2)	C9B—C10B—C14B	120.0 (2)
C2A—C3A—Cl2A	119.54 (18)	C12A—C11A—C10A	121.9 (2)
C4A—C3A—Cl2A	118.56 (17)	C12A—C11A—H11A	119.0
C2B—C3B—C4B	121.3 (3)	C10A—C11A—H11A	119.0
C2B—C3B—H3B	119.4	C10B—C11B—C12B	122.3 (2)
C4B—C3B—H3B	119.4	C10B—C11B—H11B	118.9
C3A—C4A—C5A	117.0 (2)	C12B—C11B—H11B	118.9
C3A—C4A—N1A	118.80 (18)	C13A—C12A—C11A	119.8 (2)
C5A—C4A—N1A	124.06 (19)	C13A—C12A—H12A	120.1
C3B—C4B—C5B	117.95 (19)	C11A—C12A—H12A	120.1
C3B—C4B—N1B	123.5 (2)	C13B—C12B—C11B	119.7 (2)
C5B—C4B—N1B	118.40 (19)	C13B—C12B—H12B	120.1
C6A—C5A—C4A	121.7 (2)	C11B—C12B—H12B	120.1
C6A—C5A—H5A	119.1	C12A—C13A—C8A	120.9 (2)
C4A—C5A—H5A	119.1	C12A—C13A—H13A	119.5
C6B—C5B—C4B	121.4 (2)	C8A—C13A—H13A	119.5
C6B—C5B—Cl2B	119.1 (2)	C12B—C13B—C8B	120.4 (2)
C4B—C5B—Cl2B	119.51 (15)	C12B—C13B—H13B	119.8
C5A—C6A—C1A	119.6 (2)	C8B—C13B—H13B	119.8
C5A—C6A—H6A	120.2	C10A—C14A—H10A	109.5
C1A—C6A—H6A	120.2	C10A—C14A—H10B	109.5
C1B—C6B—C5B	118.2 (2)	H10A—C14A—H10B	109.5
C1B—C6B—H6B	120.9	C10A—C14A—H10C	109.5
C5B—C6B—H6B	120.9	H10A—C14A—H10C	109.5
N1A—C7A—C8A	122.16 (18)	H10B—C14A—H10C	109.5
N1A—C7A—H7A	118.9	C10B—C14B—H10D	109.5
C8A—C7A—H7A	118.9	C10B—C14B—H10E	109.5
N1B—C7B—C8B	122.3 (2)	H10D—C14B—H10E	109.5
N1B—C7B—H7B	118.9	C10B—C14B—H10F	109.5
C8B—C7B—H7B	118.9	H10D—C14B—H10F	109.5
C9A—C8A—C13A	118.0 (2)	H10E—C14B—H10F	109.5
C9A—C8A—C7A	121.95 (18)	C7A—N1A—C4A	121.04 (17)
C13A—C8A—C7A	120.03 (19)	C7B—N1B—C4B	120.91 (19)
C9B—C8B—C13B	118.83 (19)	C9A—O1A—H1A	108.4 (19)
C9B—C8B—C7B	121.29 (19)	C9B—O1B—H1B	104 (2)
			
C6A—C1A—C2A—C3A	0.6 (4)	C13A—C8A—C9A—C10A	0.2 (3)
Cl1A—C1A—C2A—C3A	−179.00 (19)	C7A—C8A—C9A—C10A	−179.2 (2)
C6B—C1B—C2B—C3B	−0.1 (4)	C13B—C8B—C9B—O1B	−179.9 (2)
Cl1B—C1B—C2B—C3B	−177.81 (19)	C7B—C8B—C9B—O1B	0.5 (3)
C1A—C2A—C3A—C4A	0.2 (4)	C13B—C8B—C9B—C10B	1.1 (3)
C1A—C2A—C3A—Cl2A	179.75 (19)	C7B—C8B—C9B—C10B	−178.5 (2)
C1B—C2B—C3B—C4B	−0.1 (4)	O1A—C9A—C10A—C11A	179.7 (2)
C2A—C3A—C4A—C5A	−1.2 (3)	C8A—C9A—C10A—C11A	−1.4 (3)
Cl2A—C3A—C4A—C5A	179.29 (18)	O1A—C9A—C10A—C14A	−0.7 (3)
C2A—C3A—C4A—N1A	−177.4 (2)	C8A—C9A—C10A—C14A	178.3 (2)
Cl2A—C3A—C4A—N1A	3.1 (3)	O1B—C9B—C10B—C11B	179.5 (2)
C2B—C3B—C4B—C5B	0.6 (3)	C8B—C9B—C10B—C11B	−1.5 (3)
C2B—C3B—C4B—N1B	176.5 (2)	O1B—C9B—C10B—C14B	−0.8 (3)
C3A—C4A—C5A—C6A	1.4 (4)	C8B—C9B—C10B—C14B	178.2 (2)
N1A—C4A—C5A—C6A	177.4 (2)	C9A—C10A—C11A—C12A	1.3 (4)
C3B—C4B—C5B—C6B	−0.9 (3)	C14A—C10A—C11A—C12A	−178.3 (3)
N1B—C4B—C5B—C6B	−177.1 (2)	C9B—C10B—C11B—C12B	0.8 (4)
C3B—C4B—C5B—Cl2B	179.49 (18)	C14B—C10B—C11B—C12B	−178.9 (3)
N1B—C4B—C5B—Cl2B	3.3 (3)	C10A—C11A—C12A—C13A	−0.1 (4)
C4A—C5A—C6A—C1A	−0.7 (4)	C10B—C11B—C12B—C13B	0.3 (4)
C2A—C1A—C6A—C5A	−0.4 (4)	C11A—C12A—C13A—C8A	−1.1 (4)
Cl1A—C1A—C6A—C5A	179.2 (2)	C9A—C8A—C13A—C12A	1.0 (4)
C2B—C1B—C6B—C5B	−0.2 (4)	C7A—C8A—C13A—C12A	−179.6 (2)
Cl1B—C1B—C6B—C5B	177.52 (18)	C11B—C12B—C13B—C8B	−0.8 (4)
C4B—C5B—C6B—C1B	0.7 (3)	C9B—C8B—C13B—C12B	0.1 (4)
Cl2B—C5B—C6B—C1B	−179.66 (18)	C7B—C8B—C13B—C12B	179.7 (2)
N1A—C7A—C8A—C9A	−3.7 (3)	C8A—C7A—N1A—C4A	−178.4 (2)
N1A—C7A—C8A—C13A	176.9 (2)	C3A—C4A—N1A—C7A	−151.4 (2)
N1B—C7B—C8B—C9B	−3.1 (4)	C5A—C4A—N1A—C7A	32.7 (3)
N1B—C7B—C8B—C13B	177.3 (2)	C8B—C7B—N1B—C4B	−177.5 (2)
C13A—C8A—C9A—O1A	179.1 (2)	C3B—C4B—N1B—C7B	34.1 (3)
C7A—C8A—C9A—O1A	−0.3 (3)	C5B—C4B—N1B—C7B	−150.0 (2)

### Hydrogen-bond geometry (Å, °)

**Table:**

*D*—H···*A*	*D*—H	H···*A*	*D*···*A*	*D*—H···*A*
O1A—H1A···N1A	0.83 (2)	1.86 (2)	2.603 (2)	149 (3)
O1B—H1B···N1B	0.80 (2)	1.86 (2)	2.599 (2)	155 (3)
